# Pro1170 Ala polymorphism in HER2-neu is associated with risk of trastuzumab cardiotoxicity

**DOI:** 10.1186/s12885-015-1298-6

**Published:** 2015-04-11

**Authors:** Sasha E Stanton, Maureen M Ward, Paul Christos, Rachel Sanford, Christina Lam, Marta V Cobham, Diana Donovan, Ronald J Scheff, Tessa Cigler, Anne Moore, Linda T Vahdat, Maureen E Lane, Ellen Chuang

**Affiliations:** 1 Department of Medicine, Weill Cornell Medical College, 425 E 61st St. 8th floor, New York, NY 10065 USA; 2 Department of Healthcare Policy and Research, Division of Biostatistics and Epidemiology, Weill Cornell Medical College, New York, NY 10065 USA; 3 Current address: Tumor Vaccine Group, University of Washington, 850 Republican Street Box 358050, Seattle, WA 98102 USA

**Keywords:** Her2-neu, Breast cancer, Trastuzumab cardiotoxicity, Single nucleotide polymorphisms, Pro 1170 Ala polymorphism

## Abstract

**Background:**

Variations in single nucleotide polymorphisms (SNPs) have been associated with enhanced drug efficacy and toxicity in cancer therapy. SNP variations in the ErbB2 gene have been identified that alter the protein sequence of the HER2-neu protein, but how these polymorphisms affect prognosis and response to HER2 targeted therapy is unknown. We examined eleven ErbB2 SNPs that alter the HER2-neu amino acid sequence to determine whether any of these particular polymorphisms were associated with increased trastuzumab cardiotoxicity in a case–control study.

**Methods:**

140 subjects were enrolled from a single institution under Weill Cornell Medical College IRB protocol #0804009734. Patients were eligible if they had histologically or cytologically proven HER2-neu positive breast cancer and more than 3 months of trastuzumab therapy. Cases had either symptomatic CHF or a decline in LVEF of 15% (or if the LVEF <55%, a decline in LVEF of 10%) that resulted in at least temporary discontinuation of trastuzumab, whereas controls had no decline in their LVEF. Eleven ErbB2 single gene SNPs that resulted in an alteration in the HER2-neu protein amino acid sequence were studied. Single gene SNP analysis was carried out using SNP genotyping assays from genomic DNA obtained from peripheral blood or buccal swab.

**Results:**

Only two of the ErbB2 SNPs (Ile 655 Val and Pro 1170 Ala) were found to have variation. There was no association between codon 665 and cardiotoxicity; however the proline variant of amino acid 1170 was more likely than the alanine variant to be found in cases with trastuzumab cardiotoxicity (35% of case patients as compared to 17% of controls, p = 0.04). This association remained significant in multivariable analysis taking into account age, race, and history of hypertension (adjusted OR = 2.60, 95% CI = 1.02, 6.62, p = 0.046).

**Conclusions:**

The Her2/neu Pro 1170 Ala polymorphism can be used to identify a subset of patients who are at increased risk of cardiotoxicity from trastuzumab therapy. Her2/neu single nucleotide polymorphisms may be useful in conjunction with other biomarkers to risk stratify patients in order to optimize clinical management.

## Background

Variations in single nucleotide polymorphisms (SNPs) have been associated with enhanced drug efficacy and toxicity in cancer therapy [1]. The monoclonal antibody trastuzumab has provided a critical directed therapy that has dramatically improved the prognosis of 20-25% of breast cancer patients whose tumor overexpresses the HER2-neu receptor [2]. Prior to the advent of trastuzumab therapy, patients with HER2 positive tumors had significantly worse prognosis, with decreased response to chemotherapy, increased risk of relapse, and increased incidence of brain metastases compared with patients with HER2 negative tumors [3]. Although trastuzumab is well tolerated, the major toxicity is cardiomyopathy, with approximately 10% of patients developing asymptomatic left ventricular ejection fraction (LVEF) decline (defined as >10% decrease in LVEF or decline of EF to <50%) and 4% developing signs of congestive heart failure following treatment with trastuzumab as a single agent [4]. From a meta-analysis of 10 randomized control trials including 11,882 patients with early stage and metastatic breast cancer, trastuzumab therapy was associated with a 7.5% risk of LVEF decrease and a 1.9% risk of CHF. The risk of LVEF decline was 2.13-fold and CHF was 4.19-fold compared with patients not receiving trastuzumab [5]. When trastuzumab was used concurrently with anthracycline chemotherapy the risk of cardiac dysfunction was 27% compared with 13% when a non-anthracycline regimen was used [2,4]. The risk of trastuzumab cardiotoxicity was shown to be further increased in older women, women with a decreased EF prior to starting trastuzumab therapy, and women on an anti-hypertensive agent prior to starting trastuzumab [5].

The mechanism of trastuzumab cardiotoxicity is unknown. HER2/neu has been shown to be essential in cardiac myocytes in animal models. In mouse studies, knockout of the ErbB2 gene is lethal and knockout of ErbB2 in cardiac myocytes predisposes mice to dilated cardiomyopathy in response to cardiac stress (including exposure to anthracyclines) [6-8]. However, unlike the ultra-structural changes in cardiac myocytes seen with anthracyclines, including vacuolization and myocyte cell death, cardiac biopsies after trastuzumab related cardiac dysfunction showed no structural changes [9]. Whereas anthracycline toxicity is dose-dependent and irreversible, trastuzumab toxicity is not dose dependent and is reversible, and patients can often be re-treated with trastuzumab even after an initial LVEF decline if they are placed on cardiac medications [3,9,10]. Interestingly, not all targeted HER2 therapies have similar cardiotoxicity profiles, with the small molecule dual EGFR and HER2 inhibitor lapatinib having much less cardiac toxicity than trastuzumab (0.2% CHF and 1.4% asymptomatic cardiac events) [9].

Single nucleotide polymorphisms (SNPs) are normal variations in a gene sequence that may influence a drug’s efficacy or toxicity. A current review of the Exome Variant Server (http://evs.gs.washington.edu/EVS) for ERBB2 recovered 67 missense SNPs, 47 of which are believed to lead to significant protein alterations. To date the role of these SNPs on both outcomes and response to therapy in HER2 breast cancer is poorly understood [9]. The best studied is the polymorphism at amino acid 655 in the transmembrane domain of HER2-neu [11-13]. In 1039 patients with invasive HER2 positive breast cancer, the Ile 655 Val polymorphism was found to be associated with a 1.5-fold increase in HER2-neu expression and a worse outcome [14]. The Ile/Val or Val/Val genotypes have been associated with a 2-fold increase in risk of breast cancer [15]. In a recent meta-analysis of 27 case–control studies of the Ile 655 Val polymorphism, the Val/Val and Val/Ile phenotypes in patients not treated with trastuzumab (n = 816) were associated with worse disease free survival (hazard ratio [HR] = 1.5) and distant disease free survival (HR = 1.9). However, when treated with trastuzumab (n = 212) then the Val/Ile and Val/Val phenotypes were associated with better disease free and distant disease free survival [13]. In one study of 61 patients with advanced breast cancer treated with trastuzumab, there was no association between the isoform and tumor response or survival, but appeared to be an association of the valine polymorphism with cardiotoxicity [16]. From this early evidence that polymorphisms in HER2/neu amino acid sequence may identify patients that are at risk for trastuzumab cardiotoxicity, we conducted a case–control study to evaluate more comprehensively whether any of 11 identified nonsynonomous HER2/neu SNPs are associated with trastuzumab cardiotoxicity.

## Methods

### Study design

Patients were recruited from the Weill Cornell Breast Oncology group between 2007 and 2012. All patients had informed consent. This study was approved by the Institutional Review Board (IRB) of Weill Cornell Medical College under IRB protocol # 0804009734. Eligibility criteria included either histologically (IHC 2-3+) or cytologically (FISH amplification ratio ≥ 2) and history of treatment with trastuzumab (in the adjuvant, metastatic, or neoadjuvant setting). Twenty-nine of the 140 subjects, defined as the case patients, had either symptomatic CHF or a decline in LVEF of 15% (or if the LVEF <55%, a decline in LVEF of 10%) that resulted in at least temporary discontinuation of trastuzumab and controls had to have received greater than 3 months of trastuzumab therapy without a decline in their LVEF.

### Sample processing

DNA was extracted from peripheral blood mononuclear cells (PBMC) or in one case a buccal rinse. For whole blood, PBMC were separated by ficoll gradient centrifugation and PBMC DNA was extracted using phenol-chloroform extraction and ethanol precipitation. DNA pellets were dried and resuspended in Tris/EDTA (TE) buffer. For the buccal rinse, the buccal cells were pelleted and DNA was extracted using the Puregene Buccal Cell Kit following manufacturer’s instructions (Qiagen Sciences, Valencia, CA). DNA concentrations were determined using a Nano-Drop 1000 spectrophotometer (NanoDrop Products, Willmington, DE).

### SNP assay

SNP genotyping assays using validated sequence specific primers and probes from Applied BioSystems (ABI, Foster City, CA) were used to evaluate 11 known ErbB2 SNPs. Twenty nanograms of genomic DNA from each patient were assayed in duplicate for each probe and primer set following manufacturer’s instructions. Assays were performed using the ABI 7900HT real-time PCR instrument. SNP calls were made using SDS 2.4 Allelic Discrimination software (ABI, Foster City, CA). Samples were run in duplicate for every SNP reaction. Thirty-nine SNP sample PCR runs resulted in one of the two SNP PCR reactions having a failed or undetermined result. Eight SNP sample PCR runs resulted in both SNP PCR reactions having a failed result. The SNP PCR reactions having an undetermined result in both PCR reactions were repeated and a SNP call was able to be determined for every SNP for every sample. No SNP PCR runs resulted in a genotype discrepancy. The observed genotype frequencies were in Hardy-Weinberg equilibrium.

### Statistical considerations

The frequency of individual ErbB2 SNPs has not been well defined for most of the SNPs that have been identified. Sample size for the study was determined based on the assumption that the frequency of Her2-neu SNPs in controls was approximately 7.5%, which is the midpoint of the I654 prevalence and the I655V homozygote prevalence (which is in the 5-10% range). The most information exists for the I655V polymorphism, whose prevalence has been reported at between 4.9% - 10%. The prevalence of three other polymorphisms is 6.5% (I654V), 1.2% (W452C), and 10%-37% (P1170A). Assuming the frequency of the Her2/neu SNP in controls was 7.5%, by genotyping approximately 35 cardiac cases and 105 non-cardiac controls (3:1 control:case ratio), the study had 80% power (two-tailed alpha = 5%) to detect a 5-fold increase in a Her2/neu SNP genotype in cardiac cases relative to non-cardiac controls. Descriptive statistics (including mean, standard deviation, median, range, frequency, and percent) were calculated to characterize demographic and clinical factors in the case and controls. The chi-square test was used to evaluate the association between 1) SNP variant status and case/control status and 2) demographic/clinical factors (i.e., age, race, hypertension status) and case/control status. Multivariable logistic regression analysis was used to examine the association between Her2/neu SNP variant status and cardiac toxicity (i.e., cases vs. controls), after controlling for age, race, and hypertension status. Ninety-five percent confidence intervals (95% CI) for the adjusted odds ratios (OR) were calculated to assess the precision of the obtained estimates. All p-values are two-sided with statistical significance evaluated at the 0.05 alpha level. All analyses were performed using SAS Version 9.1 (SAS Institute, Inc., Cary, NC).

## Results

### Clincopathologic characteristics of the patient population

140 patients with breast cancer treated with trastuzumab were studied. There were 29 cases (patients who developed cardiotoxicity) and 111 controls (patients who did not develop cardiotoxicity). The patients included in this study had a median ejection fraction of 65% (range of 50% to 77%) prior to initiation of therapy. The cases and controls had similar hypertension history (p = 0.89) with 16% of controls and 17% of cases having a history of hypertension and the majority being normotensive (Table 1). 79% of cases and 84% of controls were Caucasian. There was a non-significant trend (p = 0.26) for cases to be of black race compared to controls (13.8% vs 5.4%).

**Table 1:** **Cases and controls are matched by age, race, and history of hypertension**

		Total	Controls	Cases
	**Number of patients**	140	111	29
**Age (years)**	Median (range)	56 (32–85)		
	<50 yo	39 (28%)	30 (27%)	9 (31%)
	>50 yo	101 (72%)	81 (73%)	20 (69%)
**Gender**	Female	140 (100%)		
**Race**	White	112 (80%)	93 (84%)	23 (79%)
	Asian	18 (13%)	12 (11%)	2 (7%)
	Black	10 (7%)	6 (5%)	4 (14%)
**Baseline LVEF**	Median (range)	65% (50-77%)		
**History of Hypertenson**	Yes	23 (16.4%)	18 (16%)	5 (17%)
	No	117 (84%)	93 (84%)	24 (83%)

### Frequency of SNP variation in population

The characteristics of the 11 HER2-neu SNPs examined are shown in Table 2. Of these 11 SNPs, only two showed variation among our study population, Rs1058808 and Rs1136201 (Table 3). Rs1136201 is a change from A to G which causes an amino acid change from isoleucine to valine at codon 655 in the transmembrane domain of the HER2-neu protein (Table 2). The AA variant (Ile/Ile) was present in 68% of the population, the AG variant (Ile/Val) in 29% of the population, and the GG (Val/Val) variant in 3% of the population (Table 3). Rs1058808 is a change from C to G that causes an amino acid change from proline to alanine at codon 1170 in the carboxyterminus of the HER2-neu protein. The CC variant (Pro/Pro) was present in 20.7% of this patient population, the CG variant (Pro/Ala) in 45.7% of the population, and the GG variant (Ala/Ala) in 33.6% of the patient population. None of the SNPs located in the kinase domain showed variation.

**Table 2:** **Location, polymorphism, and amino acid change found in eleven HER2 SNPs studied**

SNP	Polymorphism	Codon	Amino acid change	Protein domain
**Rs4252633**	G/T	452	Trp-Cys	Ligand Binding
**Rs1136201**	A/G	655	Ile-Val	Transmembrane
**Rs34602395**	G/T	703	Ser-Ile	Tyrosine Kinase
**Rs56366519**	C/T	738	Ser-Ile	Tyrosine Kinase
**Rs28933369**	A/G	776	Gly-Ser	Tyrosine Kinase
**Rs28933370**	A/G	857	Asn-Ser	Tyrosine Kinase
**Rs28933368**	A/G	914	Asp-Lys	Tyrosine Kinase
**Rs2172826**	C/G	927	Pro-Arg	Tyrosine Kinase
**Rs1058808**	C/G	1170	Pro-Ala	Carboxy terminus
**Rs55943169**	A/C	1186	Ala-Asp	Carboxy terminus
**Rs36085723**	A/G	1253	Val-Met	Carboxy terminus

**Table 3:** **Polymorphism variation in the 11 HER2 SNPs**

Rs4252633		Rs1136201		Rs34602395		Rs56366519		Rs28933369		Rs28933370		Rs28933368		Rs2172826		Rs1058808		Rs55943169		Rs36085723	
Geno	n	Geno	n	Geno	n	Geno	n	Geno	n	Geno	n	Geno	n	Geno	n	Geno	n	Geno	n	Geno	n
TT	0	AA	95	GG	140	TT	140	GG	140	AA	140	GG	140	CC	140	CC	29	CC	138	AA	0
TG	0	AG	41	GT	0	CT	0	AG	0	AG	0	AG	0	CG	0	CG	64	AC	2	AG	0
GG	140	GG	4	TT	0	CC	0	AA	0	GG	0	GG	0	GG	0	GG	47	AA	0	GG	140
**Tot**	140	**Tot**	140	**Tot**	140	**Tot**	140	**Tot**	140	**Tot**	140	**Tot**	140	**Tot**	140	**Tot**	140	**Tot**	140	**Tot**	140

Abbreviations: Geno (genotype), n (number), Tot (total).

### Association of Her2/neu polymorphisms with symptomatic cardiomyopathy in the study population

There was no association observed between the codon 655 polymorphism and cardiotoxicity (p = 0.96, data not shown). The CC (Pro/Pro) genotype at codon 1170 was associated with cardiotoxicity (34.5% of the cases (10/29) as compared to 17.1% of the controls (19/111) (p = 0.04)) (Table 4). SNP frequencies can vary by ethnicity. We only had 10 black subjects and 14 Asian subjects in our study and therefore did not carry out an association analysis for each ethnicity separately. However, we considered the question of whether a higher frequency of black subjects might express the CC (Pro/Pro) genotype, since we had found that there was a nonsignificant trend toward black ethnicity being associated with cardiotoxicity. The CC genotype occurred in 1 black subject (10% of the black population), the GC genotype occurred in 5 subjects (50%) and the GG genotype in 4 subjects (40%). From the database http://evs.gs.washington.edu/EVS for the SNP rs1058808 in African American subjects (n = 2203) the CC genotype does occur more frequently (58.6% of subjects), with the GG genotype occurring in 5.4% and the GC genotype occurred in 36% of subjects. However, within our small black population the CC genotype was not numerically increased in black subjects. In a multivariable logistic regression model for predictors of case/control status, after controlling for age, ethnicity, and previous history of hypertension, only the 1170 polymorphism significantly predicted case (cardiotoxicity) vs. control (no-cardiotoxicity) status following trastuzumab therapy (p = 0.046, adjusted OR = 2.60; 95% CI = 1.02 to 6.62) (Table 5).

**Table 4:** **Increased cases of trastuzumab cardiotoxicity with genotype Pro/Pro**

Codon 1170	Group	
	Control	Case
**Pro/Pro**	19 (17.1%)	10 (34.5%)
**Pro/Ala or Ala/Ala**	92 (82.9%)	19 (65.5%)

p = 0.04.

**Table 5:** Higher prevalence of Pro/Pro genotype in cases after **multivariate analysis**

Variables	P-value	Adjusted OR	95% CI	
			Lower	Upper
**Pro/Pro**	0.046	0.385	0.151	0.983
**Age**	0.885	0.932	0.357	2.43
**Race**	0.51	1.426	0.496	4.095
**Hypertension**	0.75	1.182	0.376	3.713

Pro/Pro (referent = Ala/Ala or Ala/Pro).

## Discussion

We studied 140 women with HER2 positive breast cancer treated with trastuzumab. Based upon predefined statistical considerations, we compared 29 cases with cardiotoxicity to 111 controls. The cases and controls were similar in age (27% of the controls and 31% of the cases under the age of 50, p = 0.67), race (predominantly Caucasian, 84% of the controls and 79% of the cases with 11% of the controls and 8% of the cases Asian, p = 0.26), history of hypertension (16% of the controls and 17% of the cases, p = 0.89), and median LVEF at the start of the study (Table 1). As hypertension, low LVEF, and age are all factors that can contribute to trastuzumab toxicity [9], the similarities of these factors between the cases and controls suggest that the trastuzumab cardiotoxicity can be associated with the SNP variants independent of these other factors. Interestingly, there was a non-significant trend suggesting that case patients may be more likely to be of black race compared to controls (Table 1, 13.8% of the cases and 5.4% of the controls). However, the small sample size is too low to make reliable inferences, and these results should be further addressed in larger studies which include a higher proportion of black subjects.

Of the 11 ErbB2 SNPs studied that caused HER2-neu protein amino acid variations, only two, corresponding to Ile 655 Val and Pro 1170 Ala, were found to have variation among the ethnically mixed population with HER2 positive breast cancer in this study. For the Ile 655 Val polymorphism (Rs1136201) there were 68% AA (Ile/Ile), 29% AG (Ile/Val), and 3% GG (Val/Val) (Table 3). These results are similar to the published results of the distribution Ile 655 Val polymorphism. For example in one study there was 72% Ile/Ile, 26% Val/Ile, and 2% Val/Val [13]. The Ile 655 Val polymorphism is located in the transmembrane domain of the HER2-neu protein (Table 2) and has been suggested to increase the dimerization of the receptor thus increasing its expression [17]. In contrast with a previous report of an association of the Ile 655 Val polymorphism with increased rates of congestive heart failure [16], in our study the Ile 655 Val polymorphism was not associated with congestive heart failure. This difference may be due to the larger sample size in our study (140 subjects with 29 cases of cardiotoxicity) compared to the previous study (61 subjects with 5 cases of cardiotoxicity), or to differences in cardiotoxicity definition or to differences in patient population characteristics.

Instead we found that the Pro 1170 Ala polymorphism was associated with trastuzumab cardiomyopathy. The Pro/Pro genotype was present more frequently in the cases (10/29 patients, 34.5%) than the controls (19/111 or 17.1%) (Table 4). This association held up in multivariable analysis when controlling for age, race, and hypertension status. The Pro/Pro genotype was independently associated with cases who developed cardiotoxicity as compared to the either the Pro/Ala or Ala/Ala genotype (adjusted OR = 2.60, p = 0.046, Table 5). However, because we also had explored additional pairwise comparisons for GG vs other (p = 0.75) and GC vs other. (p = 0.17), the alpha level should be corrected for multiple comparisons. The new multiple comparison- adjusted alpha level would be 0.017 (0.05/3). Thus, after adjustment for multiple comparisons, our result would not be significant and therefore replication of the observed findings is definitely required from future studies.

The Pro1170 Ala SNP, along with other biomarkers such as micro RNA and troponin levels [9], could ultimately be used to stratify patients into high and low risk subsets so that clinical management could be tailored to minimize the risk of cardiotoxicity. This is an important problem because when trastuzumab is discontinued due to cardiac toxicity, it deprives patients of this life-prolonging drug. Overall our study has the limitation that it is a single institution study. These results should be confirmed with larger sample sizes, preferably with inclusion of more minority subjects.

Compared with the Ile 655 Val polymorphism, the Pro 1170 Ala polymorphism has been less well studied. In the only study evaluating breast cancer and the Pro1170 Ala polymorphism that we could find, it was reported that the Pro1170 Ala polymorphism did not correlate with increased breast cancer risk [14]. Amino acid codon 1170 is located at the carboxydomain of the Her2-neu receptor and is not part of the trastuzumab binding site, the ligand binding domain, or the heterodimerization domain. The carboxydomain contains tyrosine residues that are phosphorylation sites for the kinase. Both proline and alanine have non-polar side chains but proline has a secondary amide structure which can allow more stable hydrogen binding of nearby amino acids [18]. How this could alter downstream signaling and lead to increased trastuzumab associated cardiotoxicity is unclear.

## Conclusion

The Her2/neu polymorphism Pro 1170 Ala is associated with a 2.6-fold increased risk of cardiotoxicity in patients with HER2-neu positive breast cancer treated with trastuzumab. This result suggests that Her2/neu single nucleotide polymorphisms may be useful in conjunction with other biomarkers to risk stratify patients into those who might benefit from early institution of cardiac medications or who might be considered for non-anthracycline containing chemotherapy regimens in order to avoid cardiotoxicity.

## Abbreviations

EF: Ejection fraction
LVEF: Left ventricular ejection fraction
Pro: Proline
Ala: Alanine
Val: Valine
Ile: Isoleucine
SNP: Single nucleotide polymorphisms
